# Predictors of arteriovenous fistula maturation in hemodialysis patients: a prospective cohort from an ambulatory surgical center in Joinville, Brazil

**DOI:** 10.1590/2175-8239-JBN-2022-0120en

**Published:** 2022-12-09

**Authors:** Claudete Gasparin, Helbert do Nascimento Lima, Ademar Regueira, Alexandre Gustavo Baggenstoss Marques, Gilmar Erzinger

**Affiliations:** 1 Universidade da Região de Joinville, Programa de Pós-Graduação em Saúde e Meio Ambiente, Joinville, SC, Brazil.; 2 Fundação Pró-Rim, Joinville, SC, Brazil.

**Keywords:** Renal Insufficiency, Chronic, Renal Dialysis, Arteriovenous Fistula, Ultrasonography, Insuficiência Renal Crônica, Diálise Renal, Fístula Arteriovenosa, Ultrassonografia

## Abstract

**Introduction:**

The high rate of arteriovenous fistula maturation failure is a concern in a scenario of growing numbers of patients on hemodialysis. Non-vascular factors tied to maturation success have not been fully discussed.

**Methods:**

This prospective observational cohort study included patients with CKD on dialysis or pre-dialysis prescribed arteriovenous fistula creation for the first time in an ambulatory surgical center in Joinville, Brazil, from January 2021 to July 2021. Anthropometric aspects, sociodemographic characteristics, comorbidities, and vascular parameters observed in Doppler ultrasound were analyzed. Variables associated with maturation were analyzed in multivariate models by logistic regression.

**Results:**

Eighty-eight of 145 participants (60.1%) were males. Included patients had a median age of 59 years. Successful arteriovenous fistula maturation occurred in 113 (77.9%) patients. Factors such as increased BMI, hematocrit, arm circumference, and skinfold thickness were associated with lower chances of arteriovenous fistula maturation in univariate analysis. On the other hand, larger vein and artery diameter and fistulas in the more proximal portion of the arm were associated with higher maturation success. In multivariate analysis, smoking and larger skinfold and arm circumference were associated with lower chances of successful maturation. Increased systolic blood pressure and vein diameter were associated with greater chance of success.

**Conclusion:**

In addition to the vascular parameters assessed in Doppler ultrasonography, factors related to obesity and/or nutritional aspects may influence arteriovenous fistula maturation.

## Resumo

**Introdução:**

A alta taxa de falha na maturação da fístula arteriovenosa é motivo de preocupação para o crescente número de pacientes em hemodiálise. Os fatores não vasculares não foram totalmente estudados em relação ao sucesso da maturação.

**Métodos:**

Estudo de coorte prospectivo, observacional de pacientes com DRC diálise ou pré-diálise encaminhados para a primeira criação de fístula arteriovenosa em um centro cirúrgico ambulatorial de Joinville, Brasil, de janeiro de 2021 a julho de 2021. Aspectos antropométricos, características sociodemográficas, comorbidades, além de fatores vasculares verificados pelo ultrassom Doppler. As variáveis associadas à maturação foram analisadas em modelos multivariados por regressão logística.

**Resultados:**

Dos 145 pacientes participantes, 88 (60,1%) eram homens, com idade mediana de 59 anos. Houve sucesso na maturação da fístula arteriovenosa em 113 (77,9%) pacientes. Fatores como aumento do IMC, hematócrito, circunferência do braço e valor das dobras cutâneas foram associados a menor chance de maturação da fístula arteriovenosa na análise univariada. Por outro lado, o maior diâmetro da veia e da artéria e fístulas na porção mais proximal do membro superior foram associados a maior sucesso de maturação. Na análise multivariada, tabagismo, maior dobra cutânea e circunferência do braço foram associados a menor chance de sucesso da maturação. O aumento da pressão arterial sistólica e o do diâmetro da veia foram associados a maior chance de sucesso.

**Conclusão:**

Além dos aspectos vasculares avaliados pela ultrassonografia Doppler, fatores relacionados à obesidade e/ou a aspectos nutricionais podem influenciar a maturação da fístula arteriovenosa.

## Introduction

There has been an increase in the number of patients requiring renal replacement therapy worldwide^1^. According to data from the latest Brazilian Dialysis Census, about 145,000 patients were on dialysis in Brazil in 2020, with 92.6% of them on hemodialysis^2^. Arteriovenous fistula (AVF) has been described as a less morbid vascular access to start hemodialysis^3^. However, the exact factors associated with successful AVF maturation are not fully understood.

Chronic kidney disease (CKD) is a major public health issue known for high morbidity and mortality, growing incidence in older populations, and significant negative impacts on the quality of life of patients^1,3^. Although the use of AVF in hemodialysis presents lower complication and morbidity rates when compared to venous catheters^4^, many patients do not have an AVF in their first hemodialysis session^5^. According to data from the Brazilian Society of Nephrology, the number of patients undergoing dialysis without an AVF grew considerably from 2013 to 2018 (from 15.4% to 23.6%)^6^.

Although the success rate of AVF maturation varies^7^, patency after one year has been estimated at around 60% and 51% after two years^8^. Several factors such as vessel diameter and quality, surgeon experience, and hemodynamic conditions have been associated with successful AVF maturation^9^. Doppler ultrasound to map and decide which vessels to use in creating an AVF^10–12^ has been suggested in patients at higher risk of maturation failure^4^. However, besides vascular parameters^4,12^, other clinical characteristics have not been consistently associated with successful AVF maturation in predictive models^8,13^.

Brazil ranks third in the list of nations with the largest number of dialysis patients^14^. Therefore, understanding the factors associated with higher success rates in creating an AVF is an important element in the care provided to patients on dialysis. This study aimed to evaluate the vascular and nonvascular factors associated with the maturation of the first AVF created in patients undergoing dialysis.

## Methods

### Study Design and Setting

This prospective observational cohort study included patients with CKD referred to an ambulatory surgical center in Joinville, Santa Catarina/Brazil, for AVF creation, from January 2021 to June 2021. Joinville is a city of approximately 600,000 inhabitants^15^. The referring dialysis center provides care to all patients seen through the public healthcare system in the city.

### Study Population and Inclusion and Exclusion Criteria

The study included patients aged 18 years or older with stage V CKD on dialysis or not, who were referred to an ambulatory surgical center for AVF creation.

The patients who died before AVF maturation assessment and individuals lost during follow-up were excluded. Study participants received, read, and signed an informed consent form. The Research Ethics Committee at Univille approved the study design and granted it certificate CEP CAAE 38364920.0.0000.5366.

### Variables and Procedures

A nurse at the ambulatory surgical center conducted the interviews using a structured questionnaire at the time of AVF creation. The collected data included socio-demographic variables (gender, age, race), etiology of kidney disease, associated comorbidities (smoking, diabetes mellitus, and systemic arterial hypertension), history of use of anti-platelet drugs, and history of use of a vascular catheter for hemodialysis. Body mass index (BMI) and mean blood pressure values from two measurements were also evaluated. Skinfold thickness measurements of the biceps and triceps of the arm in which the AVF was created were also taken. Skinfold thickness of the biceps was measured along the longitudinal axis of the arm, on its anterior side, at the point of greatest apparent circumference of the belly of the biceps^16^. Tricipital skinfold thickness was also measured on the posterior side of the arm, parallel to the longitudinal axis, at the point equivalent to half of the distance between the south lateral border of the acromion and the olecranon^16^. Arm circumference was measured with a tape on the forearm to assess adipose tissue^16^. Before the surgical procedure, Doppler ultrasound examination was performed in all patients to check the vascular anatomy, a routine procedure in the service. A Doppler ultrasound device (Mindray) diagnostic Ultrasound System™ model Z5, with a linear transducer with variable frequency from 7.5 to 10 MHz was used in the assessment of vascular parameters. Doppler ultrasound examination was performed in the immediate preoperative period. After placing a tourniquet on the examined arm, the cephalic, basilic, and brachial veins were evaluated for anatomical position and presence of thrombophlebitis; caliber measurements were made on cross-sections. The brachial, radial, and ulnar arteries were assessed for anatomical position, presence of stenosis or dilatation; caliber measurements were made on cross-sections^17^. After careful analysis, the best site for AVF creation was determined. Ultrasound examination was performed by a vascular surgeon. The two surgeons involved used the same technique to create the AVFs. The radiocephalic, brachiocephalic, brachiobasilic and ulnar-basilic fistulas were created with the patients on local anesthesia, atraumatic dissection of the vessels, local intravenous administration of heparin solution on a 1:100 ratio, end-to-side anastomosis with 6–0 or 7–0 polypropylene thread, and release of venous adhesions. The brachiobasilic fistulas were made in a single- or two-stage procedure.

### AVF Creation Follow-Up Care

The same nurse gave patients advice on AVF care and scheduled a maturation review appointment within four to six weeks of AVF creation. At the review appointment, the development of AVF caliber and flow were observed with Doppler ultrasound to confirm AVF maturation. A functional AVF was deemed mature when its venous branch had a diameter equal to or greater than 0.40 cm and flow velocity equal to or greater than 500 mL/min^18^.

### Statistical Analysis

Quantitative variables were evaluated based on mean values and standard deviation. Qualitative variables were evaluated in terms of frequencies and proportions. Student’s t-test was used to compare quantitative variables and the chi-squared test to compare qualitative variables between the two groups. Variables potentially associated with the outcome were assessed with univariate logistic regression. Variables related to AVF maturation with p-values < 0.200 were included in a multivariate logistic regression model. Considering the positive linear correlation existing between arm circumference and skinfold thickness (r^2^ = 0.68), we opted to prepare two multivariate models and consider each variable separately to avoid collinearity. The final models were adjusted by backward elimination. A p-value < 0.05 was considered significant. Statistical software package STATA version 10.0 was used in statistical analysis.

## Results

Five of the 150 patients initially included in the study were excluded (two died and three were lost during follow-up). Of the remaining 145 patients, 60.7% were men; their mean age was 59 years; 74.5% were white; and 57.2% were on dialysis at the start of the study. The most prevalent comorbidities were systemic arterial hypertension (97.9%) and diabetes mellitus (47.6%). Of the 145 AV fistulas created, 113 (77.9%) were considered mature and 32 (22.1%) immature after four to six weeks. The general characteristics of the study population and stratification based on having a mature or immature AVF are presented in Table 1. Patients with a mature AVF were more obese and had higher mean hematocrit and hemoglobin levels. No significant difference was found between groups for other clinical characteristics. Regarding the vascular and anatomical characteristics of the arm in which the AVF was created (Table 2), patients with a mature AVF had smaller arm circumferences and skinfold thicknesses, and larger mean diameters in the artery used in the creation of the AVF. In addition, individuals with successful maturation had the AVF created more at the level of the arm and of the brachiocephalic type, compared to individuals unable to achieve AVF maturation. In individuals with a mature AVF, the median vein diameter was 0.6 cm (interquartile range 0.5/0.8 cm; [minimum 0.4/maximum 1.0 cm]), the median artery diameter was 0.6 cm (interquartile range 0.5/0.6 cm; [minimum 0.38/maximum 0.90 cm]), the median peak systolic velocity was 93 cm/s (interquartile range 67/129 cm/s; [minimum 43/maximum 214 cm/s]), and the median blood flow was 1930 mL/min (interquartile range 1040/3365; [minimum 505/maximum 3395 mL/min]) after maturation.

**Table 1:** Characteristics of the study population in general and stratified based on AVF maturation status (N = 145)

	Total Population n = 145 (100%)		Mature AVF n = 113 (77.9%)		Immature AVF n = 32 (22.1%)		p-value
	Total or median	% or IQR	Total or median	% or IQR	Total or median	% or IQR	
Age, years	59.0	51/67	59.0	51/69	58.5	47/64	0.296
Sex, male	88.0	60.7	73.0	64.6	15.0	46.9	0.070
BMI, kg/m^2^	27.0	34/32	27.0	24/31	31.5	24/34.5	0.141
Obese (BMI ≥ 30)	52.0	35.9	35.0	31.0	17.0	53.1	0.021
SBP, mmHg	140.0	140/160	150.0	140/160	140.0	125/160	0.125
DBP, mmHg	90.0	90/100	90.0	90/100	90.0	80/100	0.495
On hemodialysis, yes	83.0	57.2	68.0	60.2	15	46.9	0.179
Etiology of CKD							0.114
DM	52.0	35.9	41.0	36.3	11.0	34.4	
SH	39.0	26.9	33.0	29.2	6.0	18.7	
PKD	18.0	12.4	10.0	8.8	8.0	25.0	
CGN	18.0	12.4	16.0	14.2	2.0	6.2	
TIN	16.0	11.0	11.0	9.7	5.0	15.6	
Undefined	2.0	1.4	2.0	1.8	0.0	0	
Hematocrit, %	30.0	26/35	30.0	26/33	32.5	29.5/38	0.017
Hemoglobin, g/dL	10.0	8/11	10.0	8/11	11.0	8.5/12	0.050
Platelets, μl × 10^3^	213.0	176/260	213.0	178/270	211.0	172/242	0.500
SH, yes	142.0	97.9	110.0	97.3	32.0	100	0.352
Diabetes, yes	69.0	47.6	54.0	47.8	15.0	46.9	0.927
Anti-platelet drugs, yes	35.0	24.1	26.0	23.0	9.0	28.1	0.550
Smoking, present or past	65.0	44.8	47.0	41.6	18.0	56.2	0.141
Previous CT on the same side of the AVF, yes	3.0	2.1	2.0	1.8	1.0	3.1	0.635

IQR = interquartile range (25^th^ percentile/75^th^ percentile); AVF = arteriovenous fistula; SH = systemic hypertension; CKD = chronic kidney disease; DM = diabetes; PKD = polycystic kidney disease; CGN = chronic glomerulonephritis; TIN = tubulointerstitial nephropathy; BMI = body mass index; CT = catheter.

**Table 2:** Anatomic and vascular characteristics of the study population in general and stratified by AVF maturation status (n = 145)

	Total Population n = 145 (100%)		Mature AVF n = 113 (77.9%)		Immature AVF n = 32 (22.1%)		p-value
	Total or median	% or IQR	Total or median	% or IQR	Total or median	% or IQR	
Arm circumference, cm	28	26/32	28	25/30	31.5	28/36	0.001
Skinfold thickness, cm	2.2	1.5/3.0	1.9	1.3/2.8	2.7	2.2/3.6	0.001
Skinfold thickness, cm	2.2	1.5/3.0	1.9	1.3/2.8	2.7	2.2/3.6	0.001
Vein pre-AVF, cm	0.4	0.3/0.4	0.4	0.3/0.4	0.3	0.3/0.4	0.093
Artery pre-AVF, cm	0.4	0.3/0.5	0.4	0.3/0.5	0.3	0.2/0.4	0.001
Side							0.873
Right	47	32.4	37	32.7	10	31.2	
Left	98	67.6	76	67.3	22	68.7	
Height							<0.001
Arm	93	64.1	81	71.7	12	37.5	
Forearm	52	35.9	32	28.3	20	62.5	
AVF							0.033
Brachiocephalic	67	46.2	57	50.4	10	31.2	
Radiocephalic	53	36.5	34	30.1	19	59.4	
Brachiobasilic	18	12.4	16	14.2	2	6.2	
Brachio-perforating	4	2.8	4	3.5	0	0
Brachio-brachial	3	2.1	2	1.8	1	3.1	

IQR = interquartile range (25^th^ percentile/75^th^ percentile); AVF = arteriovenous fistula.

Table 3 shows the univariate analysis with respect to variables associated with successful maturation. In univariate analysis, increased BMI, hematocrit, arm circumference, and skinfold thickness were associated with a lower chance of successful maturation. In contrast, patients with veins ≥ 3.6 mm in diameter, arteries ≥ 4.0 mm in diameter, or with an AVF created in the proximal portion of the arm had a higher chance of achieving AVF maturation. In multivariate analysis (Table 4), smokers and former smokers had a lower chance of AVF maturation compared with patients who never smoked, in both models. Increased systolic blood pressure and vein diameter were also associated with a higher chance of successful AVF maturation after adjustment for other variables in both models. Similarly, increased skinfold thickness and increased arm circumference were associated with a lower chance of successful AVF maturation in both models.

**Table 3:** Univariate analysis of AVF maturation predictors (n = 145)

Variables	OR	95% CI	p-value
Sex, male vs. female	2.07	0.93–4.58	0.073
Age, per year of increase	1.02	0.99–1.05	0.236
BMI, per unit of increase	0.94	0.88–0.99	0.033
Diabetes, yes vs. no	1.04	0.47–2.28	0.927
Smoking, past/present vs. never	0.55	0.25–1.22	0.144
Hematocrit, per unit of increase	0.92	0.87–0.98	0.013
Hemoglobin, per unit of increase	1.00	0.97–1.04	0.827
Platelets, per unit of increase	1.00	0.99–1.00	0.288
SBP, mmHg; per unit of increase	1.02	1.00–1.03	0.091
DBP, mmHg; per unit of increase	1.02	0.97–1.06	0.441
Anti-platelet drugs, yes vs. no	0.76	0.31–1.85	0.551
Median vein diameter, ≥ 3.6 mm vs. < 3.6 mm	2.98	1.29–6.87	0.010
Median artery diameter, ≥ 4.0 mm vs. < 4.0 mm	3.46	1.47–8.15	0.004
Arm circumference, per unit of increase	0.88	0.81–0.95	0.001
Previous CT on the same side of the AVF, yes vs. no	0.56	0.05–6.36	0.639
Site of AVF, proximal vs. distal	4.04	1.77–9.20	0.001
Skinfold thickness, per unit of increase	0.49	0.32–0.73	0.001

AVF = arteriovenous fistula; CT = catheter; SBP = systolic blood pressure; DBP = diastolic blood pressure; BMI = body mass index.

**Table 4:** Multivariate analysis of variables associated with arteriovenous fistula maturation (N = 145)

	OR	95% CI	p-value
**Model 1**			
Smoking, past/present vs. never	0.35	0.14–0.91	0.032
SBP, mmHg; per unit of increase	1.03	1.00–1.05	0.017
Median vein diameter (≥ 3.6 mm vs. < 3.6 mm)	5.22	1.89–14.37	0.001
Arm circumference, per unit of increase	0.83	0.75–0.91	< 0.001
**Model 2**			
Smoking, past/present vs. never	0.21	0.07–0.62	0.004
SBP, mmHg; per unit of increase	1.03	1.00–1.05	0.018
Median vein diameter (≥ 3.6 mm vs. < 3.6 mm)	4.89	1.77–13.51	0.002
Skinfold thickness, per unit of increase	0.32	0.19–0.56	< 0.001

SBP = systolic blood pressure.

## Discussion

The present study found a high rate of AVF maturation, with averages greater than the values described in the literature. Smoking, history of smoking, greater arm circumference or skinfold circumference were associated with a lower chance of AVF maturation. Increased systolic blood pressure and vein diameter, on the other hand, predicted successful AVF maturation.

The success rate of AVF maturation in this study (77.9%) was greater than the rates described in a meta-analysis featuring 46 studies^8^. The study population was similar to the Brazilian population on dialysis in terms of comorbidities, gender, and age^2^. We believe that part of the successful maturation of AV fistulas seen in this study may be attributed to the analysis performed prior to AVF creation to identify the best vessels and the site of the AVF based on ultrasound examination by a vascular surgeon. According to randomized controlled trials^19,20^, the use of Doppler ultrasound as an assessment method prior to AVF creation is not superior to clinical assessment. Nevertheless, several studies have shown an association between Doppler parameters and AVF maturation^12,17,21,22^. As suggested in the latest consensus on vascular access for dialysis patients^4^, the analysis of vessels for creating an AVF, when performed by a vascular surgeon, may bring more accurate and technical information, not yet fully measurable in current studies, which however may help achieve maturation.

Regarding the variables associated with AVF maturation in univariate analysis, although male patients showed a trend towards a higher rate of AVF maturation, this association did not hold in multivariate analysis. Gender has not been clearly associated with higher success rates of AVF in other studies^23,24^. Having veins and arteries with greater diameters and an AVF created in the proximal region of the arm were associated with higher chances of successful AVF maturation. Increased diameter of veins and arteries have been correlated with greater chance of maturation^23,25^. Although a minimum cutoff value for vessel diameter has not been clearly defined, the use of vessels with diameters of less than 2 mm is potentially a limiting factor for successful maturation^4^. Having a higher BMI, greater arm circumference and skinfold thickness were associated with a higher chance of AVF failure in the present analysis. A large study including more than 1,400 patients with AV fistulas found an association between higher BMI and poor AVF development^26^. In our review, we were unable to find studies in which the adiposity of the arm where the AVF was created was considered. Besides the technical difficulty of the procedure, increased arm adiposity might be associated with inflammation and risk of thrombosis^27^. The association between diabetes and lower AVF maturation rate has not been consistently reported^23,28^. Although patients with diabetes present with endothelial alterations and imbalances in vasoactive mediators^23^, the creation of an AVF in larger-caliber veins in the more proximal portions of the arm in the study population may have partly minimized the negative effects of diabetes. After adjustment for such variables in the proposed multivariate model, preoperative diameter of the largest vein and systolic blood pressure were independently associated with AVF maturation, while smoking and arm adiposity correlated with failure of maturation. In the proposed models, clinical assessment of arm circumference and/or skinfold thickness were not usually evaluated in other studies, although they may contribute in patient evaluation.

 The limitations present in this study must considered before its findings are generalized. First, successful AVF maturation was assessed only within 30 to 45 days of AVF creation. Criteria related to the ease of puncture and adequate flow during hemodialysis were not considered. Existence of observer bias in the vascular parameters assessed in Doppler ultrasonography cannot be ruled out, since the patients were not assessed by the two vascular surgeons concomitantly for agreement about measurements taken. Blood pressure measurement was recorded only at the time of AVF creation, which means we were unable to evaluate the impact of antihypertensive drugs or to analyze blood pressure levels throughout the study period. Despite these limitations, the present study has contributed with knowledge about factors related to successful AVF maturation in a population with characteristics similar to the ones seen in the Brazilian population on hemodialysis, considering parameters indicative of obesity and/or nutritional status related to the arm in which the AVF was created.

## Conclusion

Doppler ultrasonography of the vasculature of the arm performed by a vascular surgeon may improve AVF maturation rates. Larger vein diameter, absence of a history of smoking or hypertension, and smaller arm circumference and skin fold measurements may be associated with a higher chance of successful AVF maturation. Further studies are needed to correlate parameters of local adiposity to long-term success of AVF maturation.
